# Nucleic Acid Nanoparticles Redefine Traditional Regulatory Terminology: The Blurred Line between Active Pharmaceutical Ingredients and Excipients

**DOI:** 10.1021/acsnanomed.5c00070

**Published:** 2025-11-14

**Authors:** Seraphim Kozlov, Martin Panigaj, Laura Rebolledo, Hari Bhaskaran, Kirill A. Afonin

**Affiliations:** † Chemistry and Nanoscale Science Program, Department of Chemistry, 14727 University of North Carolina Charlotte, Charlotte, North Carolina 28223, United States; ‡ Department of Psychology and Neuroscience, University of North Carolina at Chapel Hill, Chapel Hill, North Carolina 28223, United States; § Cisterna Biologics, Carlsbad, California 92011, United States

**Keywords:** NANPs, API, drug delivery, excipient, nucleic acid therapies

## Abstract

Nucleic acid nanoparticles (NANPs) make up a structurally heterogeneous class of nanosized architectures that self-assemble from rationally designed oligonucleotides via canonical and noncanonical base-pairing. Over the past decade, extensive research and development have advanced NANP technologies, bringing them closer to clinical settings. Notably, several functional nucleic acid components, integral to NANPs, have already received regulatory approval for therapeutic use. The successful translation of NANPs requires a comprehensive understanding of not only their key quality attributes but also the definitions established by regulatory health agencies, as such classification helps apply an appropriate regulatory framework to ensure successful clinical translation. A critical analysis of current knowledge about NANPs in the context of regulatory definitions reveals that NANPs can serve as active pharmaceutical ingredients (APIs) and excipients and can even combine both functions simultaneously, depending on their intended therapeutic mechanism of action and formulation context. This dual-role capacity is relatively unique among pharmaceutical materials, as most current materials serve as either an API or an excipient. Moreover, the potential for conditional activation of therapeutic functions for NANPs designed to become biologically active only in specific physiological environments adds a further layer of complexity to their regulatory classification.

## Graphical abstract

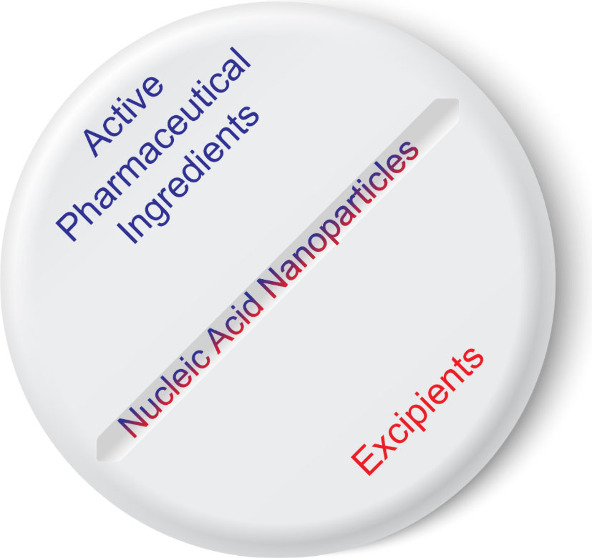

## Introduction

Nucleic acids are essential biopolymers that govern the development and function of living organisms. In addition to encoding genetic information and efficient regulation of its expression, nucleic acids act as genetic material in particles involved in horizontal gene transfer such as viruses. Due to their key and conservative roles, both DNA, as the genetic blueprint, and RNA, as a functionally versatile regulatory molecule, have been extensively explored for applications in gene therapy.

Therapeutic nucleic acids (TNAs) are synthetic oligonucleotides designed for enhanced stability, specificity, and resistance to degradation while retaining the capacity to adopt defined structures and interact with biological molecules within the cellular machinery. Engineered TNAs are used for specific gene silencing, gene editing, modulation of protein translation, and regulation of protein activities and function. Today, dozens of TNAs have received regulatory approval in the US and/or Europe. These include formulations of antisense oligonucleotides, or ASOs (e.g., Vitravene, Kynamro, Qalsody), aptamers (e.g., Macugen, Izervay), small interfering RNAs, or siRNAs (e.g., Onpattro, Leqvio), mRNAs (e.g., Comirnaty, Spikevax), and CRISPR-Cas9-based therapies (e.g., Casgevy), all of which specifically target distinct biological pathways and mechanisms.^1^

Combining different TNAs, such as aptamers and siRNAs, into a single formulation enables their simultaneous delivery and functional synchronization.^2^ Many chimeric constructs are designed to bind specific cell surface receptors via aptamers and then enter the cells to modulate post-transcriptional gene expression using linked siRNAs. The ability to assemble multiple copies or functionally distinct TNAs into self-assembled nanocomplexes has revolutionized nucleic acid technologies.

The inherent programmability of RNA and DNA molecules, along with their capacity to form intra- and intermolecular hydrogen bonds, has enabled the creation of programmable multistranded structures in a wide range of sizes, shapes, and dimensions,^3–5^ now collectively referred to as nucleic acid nanoparticles (NANPs).^6–11^ In this bottom-up approach, various naturally occurring and rationally designed RNA motifs, generated in several ways,^12^ are assembled into higher-order structures, much like fitting RNA oligonucleotides together in a molecular jigsaw puzzle (Figure 1).^10,13^ The resulting NANPs can be conjugated with cocktails of TNAs, small-molecule drugs, and imaging agents,^14–17^ enabling combinatorial and synergistic therapeutic potential.^18^

**Figure 1:**
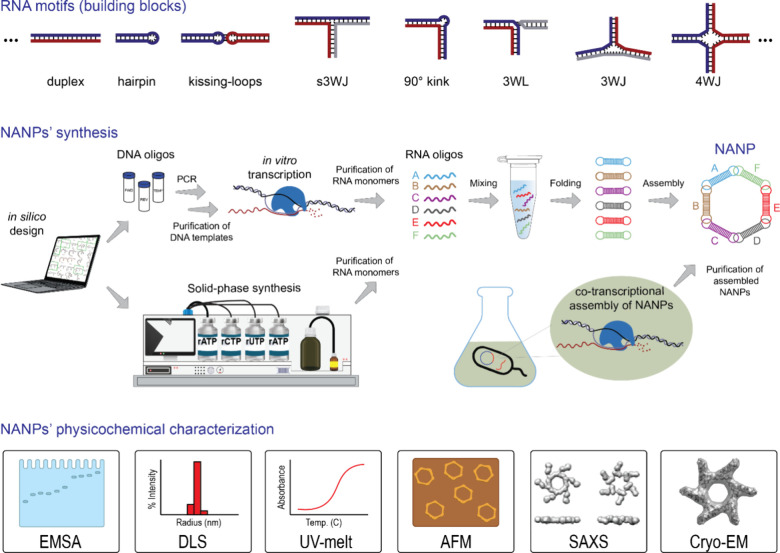
Simplified workflow of RNA-based NANPs production from simple motifs designed to higher order structures, synthesis of individual constituent strands up to their assembly, and physicochemical characterization. Constituting monomers can be synthesized by several approaches: enzymatic (*in vitro* transcription), chemical (solid phase), or biological (intracellular overexpression).^12^ The physicochemical properties of NANPs can be characterized by Electrophoretic Mobility Shift Assay (EMSA), Dynamic Light Scattering (DLS), UV Melting (UV-melt), Atomic Force Microscopy (AFM), Small-Angle X-ray Scattering (SAXS), and Cryo-Electron Microscopy (Cryo-EM).

Computational methods have become instrumental for the *de novo* design and optimization of short DNA or RNA sequences arranged into 2D and 3D NANPs structures.^19–22^ Extensive comparative studies of comprehensive libraries of NANPs^23,24^ have shown that their biological properties can be finely tuned by modifying architectural parameters such as size, shape, composition, connectivity rules, the number and orientation of functional moieties, and complexation with various lipids and polymers for intracellular delivery.^23–29^

The standard procedure for assembling NANPs typically involves a one-pot, equimolar mixture of constituent strands, followed by thermal denaturation and snap cooling, or incubation at specific temperatures to enhance intra- or interstrand annealing of complementary sequences within the oligonucleotide monomers.^30,31^ NANPs composed exclusively of RNA strands or their chemically modified analogues have also been shown to form co-transcriptionally when their corresponding DNA templates are mixed in an *in vitro* transcription reaction, incubated, and subsequently, assembled NANPs are gel-purified and further tested.^19,32,33^ More recently, a top-down strategy was introduced in which the isothermal assembly of NANPs is nuclease-driven: functionally inert double-stranded DNA/RNA hybrids are selectively digested by DNase or RNase H, yielding RNA or DNA NANPs, respectively.^30^ Such enzyme-driven approaches may enable the development of NANPs that form and become functional only after specific intracellular enzymatic processing.

In parallel, a conceptually different approach, coined as “DNA-origami”, has been developed. This is a method of folding long single-stranded DNA (ssDNA) into various shapes and patterns on a nanometer scale.^34^ DNA origami relies on the thermal annealing of long (∼7 kb) ssDNA “scaffold strands” with short complementary oligonucleotide “staples” through canonical Watson–Crick base pairing. The original DNA origami produced 2D shapes, but subsequent developments enabled both 2D and 3D DNA-origami structures.^35^ Several years after the original DNA-origami invention, hybrid RNA-DNA origami has been introduced,^36^ and more recently, fully co-transcriptionally folded RNA-origami has been developed.^8,37–40^

NANPs exhibit a broad distribution in size and molecular weight depending on their compositions and preparation methodology (Table 1). Their technological and biomedical applications span widely from structural scaffolding to the regulated administration of bioactive materials. For example, DNA and RNA/DNA origami are used for the delivery of drugs^41^ and vaccine antigens,^42^ and as biomimetic multidye systems for optoelectronic devices such as quantum information processors and solar energy converters.^43^ DNA-templated metal nanoclusters are used in diagnostic devices,^44^ as well as antimicrobial agents,^45^ and for photothermal therapy.^46^ As their programmability and structural versatility enable the precise organization, encapsulation, and conditional release of various cargos, NANPs have found their primary biomedical roles in drug delivery and as vaccine adjuvants.^8,14,15,17,42,47–50^

**Table 1:** Main Characteristics of Nanosized Materials Made of Nucleic Acids^a^

Characteristics	DNA, RNA/DNA, and RNA Origami	NANPs
Composition	DNA, RNA/DNA, RNA	DNA, DNA/RNA, RNA, chemical analogues
Mode of production	• ssDNA scaffold is folded into different shapes using numerous shorter oligonucleotide “staples” designed based on sequence complementarity.^107^	• Conventional one-pot thermal annealing, where single-stranded oligos are self-assembled into defined structures with various shapes based on sequence complementarity and architectural parameters of embedded motifs^30,31^
	• A typical source of ssDNA scaffold is the genomic DNA of an M13 bacteriophage.	• RNA NANPs can be assembled co-transcriptionally from a mixture of DNA templates within the in vitro transcription reaction1.^9,32^
	• Typical source of staples are chemical and biochemical synthesis.	• Isothermal nuclease-driven assembly: functionally inert DNA and RNA structures are selectively digested by nucleases (DNase and RNase H), followed by the isothermal self-assembly of released strands.^30^
	• RNA origami structures are folded co-transcriptionally, without the need for short oligonucleotide staples.	• Typical source of DNA and RNA oligonucleotides: chemical and biochemical synthesis via in vitro transcription
Molecular weight	∼633 kDa to 23 MDa;^108^ 1.5–5 MDa^107^	∼75–150 kDa^23^
Size	∼10–100 nm^107^	∼5–200 nm^23,30,31^ in at least one dimension, depending on the nanoparticle shape
Applications	Drug delivery,^41^ vaccines,^42^ biomimetic multidye systems for optoelectronic devices^43^	Drug delivery and vaccine adjuvants^8,14,15,17,47–50^

a The main characteristics of NANPs are summarized. The table is prepared based on refs 8, 14, 15, 17, 23, 30, 31, 41–50 , and^107–109^ .

However, despite the apparent advantages, NANP technologies still face several critical barriers that preclude their broader clinical implementation. In addition to challenges that are relatively well addressed, such as nuclease stability and immune recognition, additional challenges must be overcome to ensure the successful translation of NANPs from bench to bedside. One issue is the cost-effective scale-up of NANPs production, as methods that are efficient at the laboratory scale often prove difficult to adapt to industrial production.^51^ NANPs are typically assembled from multiple strands, produced individually via chemical synthesis, *in vitro* transcription, or cell-based expression systems, each reflecting the inherent complexity of synthesis.^12^ Alongside this, achieving batch-to-batch reproducibility is needed to meet regulatory standards for quality and consistency.

Although dehydrated NANPs may not require cold-chain storage,^52^ ensuring the long-term stability of NANPs formulations, both during storage and under physiological conditions, remains a challenge that impacts clinical usability.

Therapeutic applications of NANPs face additional hurdles, particularly, their limited capacity for targeted extrahepatic delivery to specific cells or tissues. Current therapeutic strategies rely either on the complexation of NANPs with specific delivery carriers for intracellular use^53^ or on administration of NANPs without a carrier, intended for extracellular applications.^50^ Both strategies can be enhanced through functionalization with targeting moieties, such as ligands, antibodies, or aptamers.

The choice of delivery carrier impacts NANP behavior *in vivo*, influencing stability, circulation time, biodistribution, immune recognition, cellular uptake, and compartmentalization.^25–27,50^ Consequently, detailed pharmacokinetic and toxicity studies are required for each new formulation. However, even after successful delivery and trafficking to the cytoplasm, a key unresolved question remains: how long do NANPs preserve their structural integrity and functional activity within the intracellular environment?

Addressing these technical and biological challenges, in parallel with continued innovation, will be essential for translating the unique properties of NANPs into safe, effective, and regulatory-compliant therapeutics.

During the first two decades following the invention of NANPs, research efforts have been primarily focused on refining their design and production strategies. More recently, these versatile materials have advanced toward clinical translation, offering a wealth of therapeutic opportunities alongside significant translational challenges.^6,54^ Consequently, a deep understanding of the pharmaceutical quality of NANPs is an essential step toward their successful clinical application.

The quality by design (QbD) concept is frequently used in pharmaceutical and regulatory sciences to refer to the drug product development approach wherein quality is incorporated into the product design.^55^ This approach relies on understanding the key parameters, the so-called “critical quality attributes” and process parameters, that make a drug product safe and efficacious.^55^ Controlling these attributes and parameters can ensure high quality of a drug product. Nucleic acids’ high programmability and well-established production protocols make NANP technology ideal for applying the QbD approach.

The pharmaceutical quality has been described using the following equation: “Pharmaceutical Quality = *f* (drug substance, excipients, manufacturing, packaging)”.^56^ To apply this concept to NANPs, it is important to distinguish between a drug substance and an excipient. Here, we review the terminology used by regulatory agencies in the US and other countries to differentiate drug substances from excipients and analyze the literature on NANP applications in pharmaceutical products to determine whether NANPs should be classified as active pharmaceutical ingredients or excipients.

### Regulatory Terminology

The meaning of active pharmaceutical ingredient (API) and excipient is consistent between guidance documents published by regulatory agencies in North America, South America, Asia, and Australia, in that APIs are generally considered active components of formulations; in contrast, excipients refer to inactive components (Table 2). However, some variations in terminology are noted. For example, the US Food and Drug Administration (FDA), the Brazilian Health Regulatory Agency (ANVISA), and the National Medical Products Administration of China use the term “active pharmaceutical ingredient”, or API.^57–60^ The US FDA also uses the term “bulk drug substance” as a synonym for API.^57^ The European Medicines Agency (EMA), Health Canada, and the Therapeutic Goods Administration (TGA) of Australia use a shorter definition of “active ingredient”.^61–63^ Health Canada also uses API and “drug substance” as synonyms for “active ingredient”.^62,64^ The Pharmaceuticals and Medical Devices Agency of Japan uses the term “drug” to refer to the active component.^65,66^ In contrast, the Brazilian Health Regulatory Agency defines a drug as “*the pharmaceutical product, technically obtained or prepared, which contains one or more drugs and other substances, with a prophylactic, curative, palliative or diagnostic purpose*”.^58^

**Table 2:** Summary of Definitions Regulatory Health Authorities Use to Distinguish Active and Inactive Ingredients in Drug Products^a^

		Definitions	
Regulatory Agency	Ref	API	Excipient
US Food and Drug Administration, USA	^57,59,67^	“**Active pharmaceutical ingredient** *is any substance that is intended for incorporation into a finished drug product and is intended to furnish pharmacological activity or other direct effect in the diagnosis, cure, mitigation, treatment, or prevention of disease, or to affect the structure or any function of the body.”*	*“* **Inactive ingredient** *means any component other than an active ingredient.”*
		**“Bulk drug substance** *means the same as an active pharmaceutical ingredient.″*	
		**API** “*does not include intermediates used in the synthesis of the substance.”*	
European Medicines Agency, European Union and European Economic Area	^61,68^	*“Any substance or mixture of substances intended to be used in the manufacture of a medicinal product and that, when used in the production of a drug, becomes an* **active ingredient** *of the medicinal product. Such substances are intended to furnish pharmacological activity or other direct effect in the diagnosis, cure, mitigation, treatment, or prevention of disease or to affect the structure and function of the body.”*	*“An* **excipient** *is a constituent of a medicine other than the active substance, added in the formulation for a specific purpose. “*
Health Canada, Canada	^62,64^	*“* **Active ingredients** *are the substances in drugs that are responsible for the beneficial health effects experienced by consumers. The active ingredient in a pharmaceutical drug is called an active pharmaceutical ingredient (API).”*	**“Non-medicinal ingredient** *means a substance – other than the pharmacologically active drug – that is added during the manufacturing process and that is present in the finished drug product.”*
		**“Active ingredient** *means a drug that, when used as the raw material in the fabrication of a drug in dosage form, provides its intended effect.”*	
		*“* **Active pharmaceutical ingredient (API)** *(or* **drug substance** *) means an active ingredient that is used in the fabrication of a pharmaceutical. ... the terms “drug substance” and “active pharmaceutical ingredient” are considered interchangeable.”*	
Therapeutic Goods Administration, Australia	^63^	*“An* **active ingredient** *is a therapeutically active component in a products’ final formulation.”*	*“An* **excipient ingredient** *is not therapeutically active in a products’ final formulation.”*
Brazilian Health Regulatory Agency (Agência Nacional de Vigilância Sanitária (Anvisa)), Brazil^b^	^58^	*“* **Active pharmaceutical ingredient** *is an active chemical substance, medicine, drug or raw material that has pharmacological properties with a medicinal purpose used for diagnosis, relief or treatment, used to modify or explore physiological systems or pathological states for the benefit of the person to whom it is administered.”*	*“* **Excipient gas** *is any component gas, which is not an active substance, intentionally added to the formulation of a gas mixture.”*
			*“* **Adjuvant substance** *is the specific purpose substance added to injectable preparations. This substance must be selected to increase the stability of the product; not cause interference with the therapeutic efficacy or with the active ingredient assay; or cause toxicity in the dose administered to the patient. The adjuvant substance can be solubilizing; antioxidant; chelating agent; buffer; antibacterial agent; antifungal agent; antifoaming agent and others, when specified in the individual monograph.”*
Pharmaceuticals and Medical Devices Agency, Japan^b^	^65,66^	*“The term “* **drugs** *” refers to the following substances: 1) Substances listed in the Japanese Pharmacopoeia. 2) Substances (other than quasi-drugs and regenerative medicine products), which are intended for use in the diagnosis, treatment, or prevention of disease in humans or animals, and which are not equipment or instruments, including dental materials, medical supplies, sanitary materials, and programs. 3) Substances (other than quasi-drugs, cosmetics or regenerative medicine products) which are intended to affect the structure or functions of the body of humans or animals, and which are not equipment or instruments.”*	*“* **Pharmaceutical Excipients** *are substances other than active substances (API) contained in preparations. The excipients must be pharmacologically inactive and harmless in the administered amount and must not interfere with the therapeutic efficacy of the formulation. Excipients should be inactive, but are not limited to “inert diluents”. Excipients are essential for enhancing the manufacturability, stability, and bioavailability of the API”*
National Medical Products Administration, China^b^	^60^	*“API (* **Active Pharmaceutical Ingredient** *) means the active ingredient which is contained in medicine.”*	*“* **Pharmaceutical excipients** *are substances other than the active pharmaceutical ingredient (API) that have been appropriately evaluated for safety and are intentionally included in a drug delivery system.”*

a The definitions are reproduced verbatim from the references shown to allow side-by-side comparison. *English translation versions of regulatory publications from these agencies were included in the table.

b English translation versions of regulatory publications from these agencies were included in the table.

A similar variation is noted in terminology for excipients: the FDA and US Pharmacopoeia call them “inactive ingredients”,^59,67^ EMA – “excipients”,^61,68^ Health Canada – “non-medicinal ingredient”,^62,64^ Australian TGA – “excipient ingredient”,^63^ Pharmaceuticals and Medical Devices Agency in Japan, and the National Medical Products Administration of China – “pharmaceutical excipient”.^60,65,66^ The EMA recognizes that “*while most excipients are considered inactive, some can have a known action or effect in certain circumstances”*.^68^ Similarly, regulatory scientists in Japan explicitly mention that excipients “*should be inactive, but are not limited to “inert diluents”*.^65^ The Brazilian Health Regulatory Agency (Anvisa) does not have a separate definition of an excipient. Inactive substances in Anvisa guidance are defined as “excipient gas” for gases or “adjuvant substance” for buffers, stabilizers, emulsifiers, and other inactive components commonly added to drug products.^58^

Understanding the commonalities and nuances of these terms helps navigate the regulatory landscape and find relevant documents to aid in the clinical translation of new pharmaceutical products in different countries. The available research data, which clarify whether NANPs possess properties of APIs or excipients, are discussed further below.

### NANPs as APIs

NANPs designed to have various connectivity, such as cubes, rings, triangles, squares, pentagons, hexagons, and fibers, were shown to behave as potent immunological adjuvants and induce cytokine responses after complexation with lipid or polymeric carriers, which enabled their intracellular delivery (Figure 2A).^23,69–72^ The cytokine type depended on the type of carrier: interferons were seen with lipid-based carrier-delivered NANPs, and pro-inflammatory cytokines were detected when the same NANPs were delivered using a polymeric carrier.^28,73^ The magnitude of the cytokine responses depended on the NANPs’ physicochemical properties: shape (RNA cubes induced higher cytokine levels than RNA rings and RNA fibers), composition (RNA cubes were more potent than DNA cubes), and size (hexagons were more powerful than pentagons, squares, and triangles).^23^ In addition, the chemical composition of NANPs (e.g., RNA, DNA, or 2’F RNA) influences their subcellular compartmentalization and the degree of immune recognition.^27^ In these studies, lipid and polymeric carriers were inert components intended for the intracellular delivery of NANPs. In contrast, the biological activity in the form of immune cell activation and cytokine secretion was due to the NANPs.

**Figure 2:**
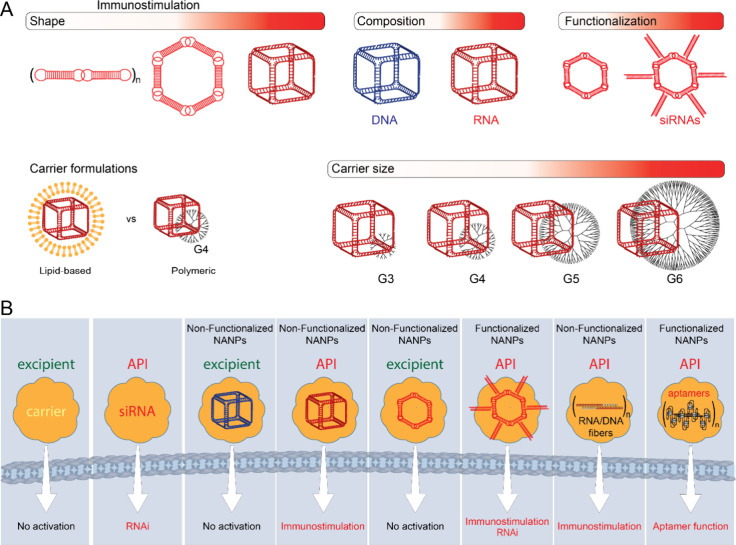
NANPs are designed to have various connectivity, such as cubes, rings, triangles, squares, pentagons, hexagons, and fibers have distinct immunostimulatory properties by themselves and by complexation with lipid or polymeric carriers (A). Nucleic acids have intrinsic bioactivity, endowing them simultaneously with excipient and API properties (B).

To better understand the mechanisms underlying NANPs’ immunostimulatory activities, it is essential to consider how the innate immune system recognizes “non-self” molecular structures. The innate immune system detects pathogen-associated molecular patterns (PAMPs), which are produced by pathogens and include proteins, lipids, carbohydrates, and nucleic acids such as viral double-stranded (ds) and single-stranded (ss) RNAs and DNAs.^74^ These PAMPs are recognized by pattern recognition receptors (PRRs), which are expressed by most cells. In addition to PAMPs, PRRs also detect damage-associated molecular patterns (DAMPs) released from stressed or dying cells.^75^ Some examples of nucleic acid sensing PRRs include cytosolic RIG-I-like receptors (RIG-I, MDA5, LGP2),^76^ endosomal Toll-like receptors (TLR3, TLR7, TLR8, TLR9),^77^ cytosolic DNA sensors (CDSs),^78^ and inflammasome-forming NLRP1.^79^ TLR3 recognizes viral dsRNA, TLR7/8 detects ssRNAs, and TLR9 senses unmethylated CpG DNA, signaling mainly via MyD88, except TLR3, which uses TRIF. RIG-I detects short RNAs with a 5′-triphosphate, distinguishing viral from host transcripts and playing a central role in antiviral defense. This sophisticated network of nucleic acid recognition provides an important understanding of how NANPs can be designed to either activate or evade innate immune recognition (Figure 3).^69,80^

**Figure 3:**
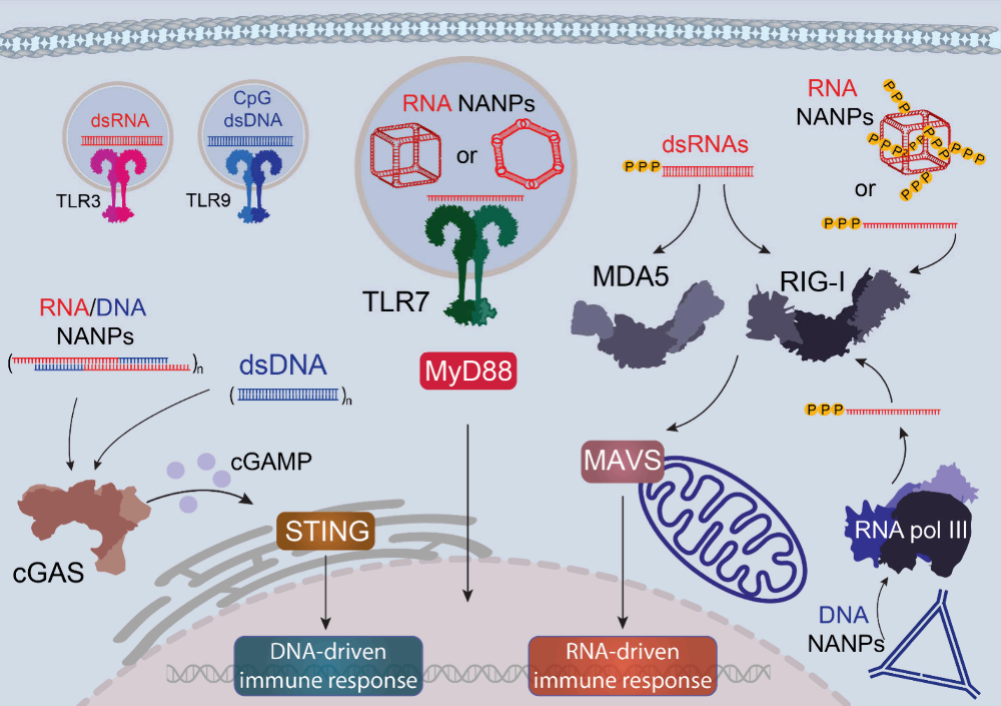
Innate immune recognition of nucleic acid nanoparticles (NANPs) is mediated by pattern recognition receptors (PRRs) that discriminate between self and non-self nucleic acids. The initial detection occurs through Toll-like receptors (TLRs), which sense pathogen-associated nucleic acid motifs within endosomal compartments. In the cytosol, RNA species are recognized by RIG-I-like receptors (RLRs), including RIG-I and MDA5, while cytosolic DNA is detected by DNA-sensing systems, such as the cGAS-cGAMP-STING pathway.

Cytokine induction by adjuvants is commonly used in vaccines to improve the immune response against antigens. Adjuvants approved by the FDA for use in current vaccines include both single-component materials, such as aluminum hydroxide (alum) and CpG DNA oligonucleotide CpG1018, and more complex mixtures (e.g., MF59, Matrix M, AS01, AS03, AS04) containing liposomes, saponin, squalene, or monophosphoryl lipid A (MPL) in addition to other immunostimulatory components.^81^ Unlike CpG oligonucleotides (e.g., ODN2216), which activate immune cells without additional components, NANPs induce cytokines only after complexation with lipid or polymeric carriers.^23,73^ Therefore, these studies provide additional examples of NANPs behave as APIs and position NANPs as safe, programmable adjuvants for next-generation cancer and infectious disease immunotherapies.

### Immunomodulation and Quality Control Considerations for NANPs for the Applications of NANPs as APIs

The therapeutic efficacy of NANPs, particularly in immunomodulatory applications, may depend on their molecular homogeneity and the absence of product-related impurities. Contaminants such as ss- and dsRNAs, truncated products, and other length impurities can disrupt NANP folding patterns, reduce stability, and increase the probability of unintended immune activation.^82–84^ In the context of cancer neoantigen vaccines, one limitation of modified-uridine mRNA is its reduced ability to elicit a sufficient cellular immune response;^85^ whereas unmodified mRNAs have been shown to engage the innate immune sensors more robustly, potentially generating more optimal responses to mRNA-encoded neoantigens.^86,87^ For the two authorized COVID-19 mRNA vaccines, much of the innate immune activation is attributed to the lipid nanoparticle (LNP) carriers, as the mRNA itself is chemically modified with N1-methylpseudouridine to dampen innate immune activation. While such modifications improve translation efficiency and reduce inflammatory toxicity, they also attenuate beneficial stimulation, as in the case of cancer vaccines; nonetheless, residual stimulation can still arise from the mRNA backbone itself. These observations highlight both the importance and the opportunity for NANPs to be produced with precisely controlled composition, defined shape and size, well-characterized immunostimulatory properties, and minimal side products, enabling the selective and predictable modulation of immune responses without inhibition of antigenic expression. Such precision could be leveraged to amplify immunity for vaccine and cancer immunotherapy applications or suppress it for inflammatory and autoimmune conditions.^88^ Future NANPs could be engineered to drive a strong cytotoxic T cell (CTL) response without interfering with a codelivered or postdelivered mRNA neoantigen vaccine. This may be achieved by systematically screening NANP libraries for candidates that preferentially induce proimmunomodulatory cytokines while avoiding excessive pro-inflammatory responses, with dose-dependence as a key control parameter. Indeed, coformulation of a KRAS mutant mRNA vaccine with cGAMP, a messenger molecule in the cGAS-STING pathway, has been shown to significantly reduce pancreatic cancer metastasis, underscoring the potential of such combinations in cancer immunotherapy.^89^ Finally, durable anticancer effects may require combining immunostimulatory NANPs and neo-antigen expressing mRNA vaccines with checkpoint inhibitors, a strategy already shown to improve clinical outcomes.^87,90^ However, some findings suggest that not all NANPs meet the definition of an API. Therefore, considering the full spectrum of available data on NANPs is essential to fully appreciate the complexity and versatility of this platform and accurately classify NANPs used in therapeutic applications.

### NANPs as Excipients

In addition to acting as APIs, NANPs have been successfully utilized as excipients to deliver various biologics, small molecules, silver ions, and therapeutic nucleic acids. For example, the receptor-binding domain (RBD) of the SARS-CoV-2 spike protein was attached to icosahedral DNA NANPs to create virus-like particles (VLPs). These were produced in two forms: monovalent (one RBD per VLP) and multivalent (6 or 30 RBDs per VLP).^42^ When tested *in vitro*, using an engineered Ramos B cell line expressing surface anti-RBD antibodies, these multivalent VLPs activated B-cell receptor signaling at the same antigen concentration that monomeric RBD did not.^42^ This *in vitro* activity correlated with *in vivo* data, demonstrating the induction of RBD-specific IgG and pseudovirus neutralization activity of VLPs.^42^ This study also reported greater potency of multivalent VLPs containing 30 RBD antigens than monovalent VLPs and VLPs containing 6 RBD antigens.^42^

RNA NANPs functionalized with EpCAM aptamers were designed to deliver SN-38, the active metabolite of irinotecan. This construct, termed 4WJ-SN38-EpCAM, inhibited tumor growth in a colorectal cancer lung metastasis model.^15^ The same 4WJ RNA platform was also successfully used to deliver the anticancer drug paclitaxel. The 3WJ and 4WJ RNA NANPs were explored to deliver paclitaxel, and it was reported that while both concepts effectively inhibit breast cancer, 4WJ NANPs have greater drug-loading capacity.^91^ Specifically, one 3WJ NANPs delivers eight paclitaxel molecules, whereas 4WJ NANPs deliver 24 drug molecules.^91^ The same study reported 32,000-fold higher paclitaxel solubility and reduced toxicity when RNA nanoparticles are used for drug delivery.^91^ NANPs designed to deliver multiple RNAi inducers for combinatorial treatment against human immunodeficiency virus effectively protected human cells from viral infection in an *in vitro* proof-of-concept experiment.^18,92^ While these examples demonstrate the potential of NANPs as excipients, other reports suggest their dual functionality, where NANPs serve both as API and as excipient.

### Simultaneous Duality

Spatiotemporal recognition of nucleic acids in cells plays a crucial role in the innate cellular immune system.^27,93^ DNA, together with RNA, serves as a key molecule in the detection of pathogens. Therefore, nucleic acids have intrinsic bioactivity, which means that NANP, designed as an excipient to deliver a drug, may still possess immunostimulatory (API) properties, leading to unintended side effects and complicating the product’s safety profile (Figure 2B). However, various modifications introduced to constituent NANP’s strands allow for tunable physicochemical and immunostimulatory properties as could be demonstrated by the following examples: (i) replacing RNA with chemical analogues increases NANPs’ thermodynamic stability and resistance to nucleases thus allowing for longer circulation and exposure to the PRRs; (ii) 3D RNA NANPs are more immunostimulatory and can activate TLR7 and RIG-I pathways, whereas 3D DNA NANPs induce minimal activation of immune cells; (iii) chemical modification (e.g., 2’F) of RNA NANPs decreases immunostimulation and limit their immunorecognition to RIG-I pathway; (iv) inclusion of 2’F modified RNAs into DNA NANPs enhances the activation of RIG-I pathway; (v) modification of RNA nucleosides, such as pseudouridine, m5C, m5U or m6A, reduces immunostimulatory properties of RNA.^27,94,95^ Future research and development must address context-dependent classification, reflecting the possibility that designed NANPs may exhibit both API and excipient properties simultaneously, regardless of their intended purpose. This simultaneous duality suggests that labeling these NANPs into one of two traditional categories, such as APIs or excipients, is not only complicated but also imperfect. In this context, a revised classification system is needed for regulatory approaches concerning a new category of structural materials with biological activity.

The regulatory situation may be complicated by the selected delivery method. For example, the naked NANPs, i.e., without any delivery agent, are poorly internalized.^23^ It seems that at least cytosolic sensing of RNA molecules depends on actin cytoskeleton remodeling that primes RIG-I-like receptor activation.^96^ Cytoskeleton disturbance is achieved by transfection of reagents that require cellular uptake and fusion. While the carrier by itself does not activate the IFN response, different delivery agents have various transfection efficiencies for the same NANP, quantitatively influencing the immune response.^23,27,26^ In addition to the magnitude of the cytokine response to NANPs delivered by different carriers, the spectrum of expressed cytokines may also differ.^73^

Interestingly, regulatory agencies (e.g., FDA, EMA) can consider lipid components differently.^97^ Based on the study of three lipid nanoparticles (Spikevax, Onpattro, and Comirnaty), it is noticed that in the case of Spikevax, lipids were considered by the FDA as part of the drug substance, while similar lipids in Onpattro and Comirnaty were reviewed as excipients. In contrast, the EMA reviewed lipids of all three LNPs as excipients. As emphasized, this comparison was made on publicly available information and does not evaluate possible proprietary data.^97^

The dual functionality of NANPs, acting simultaneously as active pharmaceutical ingredients and excipients, highlights the limitations of the traditional regulatory categories. The immune response elicited by NANPs is determined not only by their sequence or structure but also by the delivery method, carrier composition, and cellular context. As such, preclinical evaluation and regulatory assessment must consider both intrinsic bioactivity and extrinsic factors that modulate immunogenicity. Establishing standardized assays that quantify both the magnitude and spectrum of cytokine responses alongside context-specific guidelines for carrier selection could allow safer and more predictable therapeutic applications. Ultimately, a revised classification framework that acknowledges this context-dependent duality will be essential to guide the development, approval, and clinical use of structurally bioactive nucleic acid materials.

### NANPs for Conditional Activation of API

Conditionally activated NANPs (CA-NANPs) represent a major advancement in nanomedicine and an additional regulatory challenge. These materials enable precise control over the timing and location of the therapeutic activity. These smart nanostructures are programmed to respond to diverse physicochemical and biological cues, such as shifts in pH, the presence of specific RNAs or intracellular proteins, receptor engagement, or elevated concentrations of secreted or membrane-bound ligands.^98–100^ This allows for highly targeted and controlled uptake and release of disease-specific API. Therefore, the NANPs-mediated conditional activation of APIs adds further to the complexity of their regulatory classification as either excipients or APIs.

CA-NANPs can be engineered to function at all levels of biological organization, both intra- and extracellularly. Among these, the blood system represents the most accessible and critical target for a systemic approach. The ON/OFF regulation of blood coagulation by CA-NANPs exemplifies conditional activation at this level. Fibrous CA-NANPs, incorporating multiple thrombin-binding aptamers, can reversibly modulate coagulation by implementing a “kill-switch” mechanism. The increased molecular weight of the fibers, when compared to free aptamers, enhances NANP stability in blood and prolongs *in vivo* retention, as demonstrated in animal models. Upon administration of complementary “kill-switch” constructs, aptamer binding is deactivated, anticoagulant function is reversed, and the CA-NANPs disassemble into low-molecular-weight functionally inert duplexes that are rapidly cleared via the kidneys (Figure 4A).^50^

**Figure 4:**
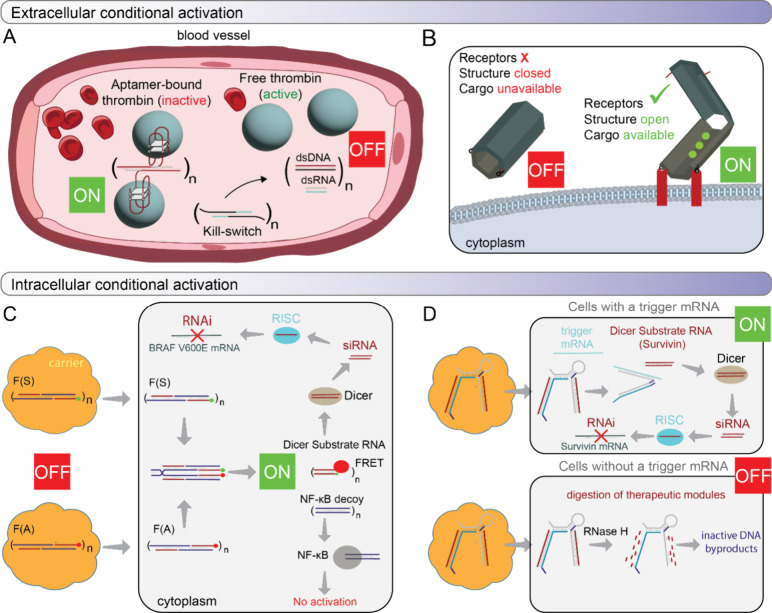
Operation of CA-NANPs at all levels of biological organization extracellularly, in body fluids (Blood) (A) and in the cell membrane (B), and intracellularly. Intracellularly, CA-NANPs can be activated as a cognate pair, simultaneously delivered to the same cell (C), or CA-NANPs’ function is triggered by an endogenous activator/transcript (D).

The cell surface of either circulating or sessile cells provides a microenvironment for cell-specific membrane receptors to sense CA-NANPs. Several interesting concepts of DNA origami-based “nanorobots” have been developed for targeted cell delivery and cargo release in cell culture and *in vivo*. Nanorobot, resembling a hexagonal barrel with two halves connected by single-stranded DNA hinges, uses two aptamers to stay locked. The barrel opens only when pairwise combinations of aptamers against platelet-derived growth factor (PDGF), TE17, or sgc8c target proteins simultaneously.^101,102^ Similarly, an autonomous hollow tube-shaped thrombin-loaded DNA nanorobot, designed via DNA origami, targets and destroys tumors *in vivo*. Detection of tumor-associated nucleolin in endothelial cells triggers a conformational change that exposes thrombin, causing tumor thrombosis and growth inhibition (Figure 4B).^103^

Intracellularly, at the genetic level, conditional activation often relies on the toehold interactions. Conceptually, this occurs either between two complementary, inactive NANPs that are introduced separately into the same cell or between the delivered nucleic acid and a specific cellular transcript. Strand displacements and isothermal reassociation release sequences to subsequently assemble a functional TNA. The mutual intracellular interaction between two hybrid NANPs can result in the activation of several split functionalities. The released RNAs can reassociate RNAi inducers, and DNA strands can form dsDNA that decoy transcription factors, preventing their relocation to the nucleus (nuclear factor kappa-light-chain enhancer of activated B cells, NF-κB) while providing a fluorescent response through FRET (Figure 4C).^104^ Recently, we introduced reconfigurable NANPs (recNANPs), engineered to specifically recognize overexpressed biomarkers and conditionally release TNAs within diseased cells only. RecNANPs demonstrate extended therapeutic effects, are nonimmunostimulatory, and can be synergistically combined with chemotherapy, offering a modular, biocompatible platform for targeted intracellular activation of TNAs (Figure 4D).^105^

## Practical Challenges and Translational Prospects

Some translational challenges and solutions learned from traditional TNAs can be applied to NANPs. For example, procedures optimized for upstream and downstream production, purification, and characterization of conventional oligonucleotides could be used to ensure high quality of oligonucleotides used to assemble NANPs.^106^

Since the immune responses elicited by NANPs are not solely determined by their sequence or structure, the delivery method, carrier composition, and target cells must be considered in the context of the intended mechanism of action. As such, preclinical evaluation and regulatory assessment must consider both intrinsic bioactivity and extrinsic factors that modulate immunological properties of NANPs. Establishing standardized assays that quantify both the magnitude and spectrum of cytokine responses, alongside context-specific guidelines for carrier selection, could allow safer and more predictable therapeutic applications. Understanding potential off-target effects and undesirable reactions to NANPs is equally important for the comprehensive evaluation of NANPs technology.

## Summary and Conclusion

NANPs have emerged as a versatile and promising class of next-generation TNAs. The current literature demonstrates that NANPs’ role in a pharmaceutical product depends on the context, design, and intended mechanism of action. Therefore, NANPs can function either as APIs or as excipients and, as discussed above, in some cases may simultaneously exhibit both roles (Figure 5). When used as APIs, NANPs typically require a delivery carrier and can induce immunostimulatory activity, which can be harnessed for applications such as vaccine adjuvants. In this context, the NANP itself is not a scaffold but the primary effector molecule (assembly), central determinant of therapeutic activity and safety, and its pharmacodynamics, pharmacokinetics, and safety profiles are subject to the same examination as other pharmaceutical products. In this role, the NANP’s sequence composition, secondary and tertiary structure, and physicochemical properties (such as size, charge distribution, and stability) collectively determine its pharmacodynamics and pharmacokinetics. Important characterization studies for NANPs include understanding of the dose–response relationships, mechanism of action, biodistribution, metabolic stability, clearance pathways, and potential off-target or immunostimulatory effects. Toxicological assessments must also address innate immune activation (e.g., via TLRs, RIG-I, cGAS), cytokine release, and organ-specific accumulation.

**Figure 5:**
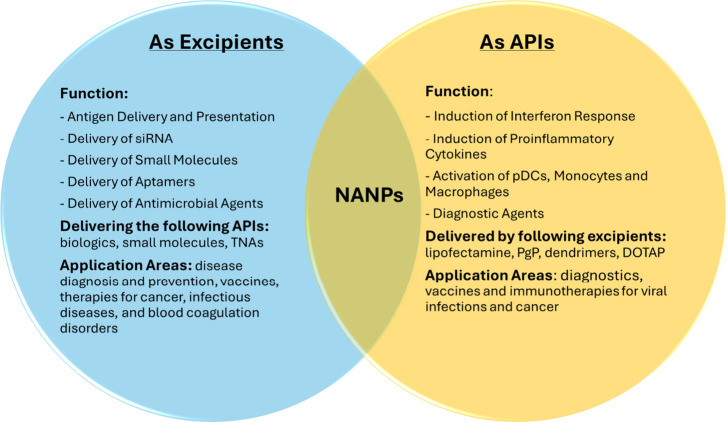
NANPs as excipients and APIs. Nucleic acid nanoparticles (NANPs) have the unique property of functioning as both excipients and active pharmaceutical ingredients (API), depending on the formulation context. Abbreviations: TNAs, therapeutic nucleic acids; pDCs, plasmacytoid dendritic cells; PgG, poly­(lactide-*co*-glycolide)-*graft*-polyethylenimine; DOTAP, 1,2-dioleoyl-3-trimethylammonium propane.

As excipients, NANPs serve as efficient supporting platforms, facilitating the stability, delivery, or bioavailability of a range of traditional therapeutics, including siRNAs, aptamers, decoys, small molecule drugs, and antigens, enabling broad applications across fields such as cancer therapy, infectious disease, and thrombosis. Here, their role is supplementary and supportive, influencing formulation properties (e.g., protection from nuclease degradation, cellular uptake efficiency, or controlled release kinetics) without contributing to independent pharmacological activity.

The intrinsic bioactivity of nucleic acids serves as a key to their recognition by the innate immune system. As a result, NANPs engineered as supporting scaffolds may still trigger immunostimulatory responses depending on their context and design, complicating their safety profiles and regulatory classification.

Potential for dual activity, whether intentional or unintentional, is a unique feature of NANPs, which distinguishes them from most traditional pharmaceutical components. This dual functionality uses a more nuanced risk-benefit assessment during development, as the conventional binary classification of API versus excipient does not fully capture their complexity. The biological activity of NANPs can be further finely tuned through chemical and structural modifications.

Moreover, the choice of delivery agent can significantly influence NANP behavior in biological systems, further challenging safety and efficacy evaluation. Transfection reagents and nanocarriers facilitate delivery by promoting cellular entry and endosomal escape but also influence the magnitude and profile of cytokine responses. Different carriers can trigger distinct immune signatures even for the same NANP, highlighting the context-dependent nature of the immune responses. Overall, the dual functionality of NANPs challenges traditional distinctions between APIs and excipients. Their biological activity is determined not only by sequence or structure but also by delivery system, carrier composition, and cellular environment. To ensure safe and predictable therapeutic outcomes, preclinical and regulatory evaluations must integrate both intrinsic bioactivity and extrinsic modulators of the immune response.

In conclusion, these considerations highlight the need for updated frameworks in both research and regulatory contexts. Rather than attempts to fit NANPs into the traditional API/excipient dichotomy, future strategies should acknowledge their multifunctional and context-dependent properties. Such an approach will be essential to guide the safe and effective translation of NANPs from the laboratory to clinical applications, allowing their full therapeutic potential while carefully managing immunological and safety considerations.
